# Laser-Induced Silver Seeding on Filter Paper for Selective Electroless Copper Plating

**DOI:** 10.3390/ma11081348

**Published:** 2018-08-03

**Authors:** Chang-Chun Liu, Jin Cheng, Xiao-Qiang Li, Zhi-Jie Gu, Kenji Ogino

**Affiliations:** 1 Graduate School of Bio-Applications and Systems Engineering, Tokyo University of Agriculture and Technology, Koganei, Tokyo 184-8588, Japan; s167438z@st.go.tuat.ac.jp; 2 College of Chemistry and Materials Engineering, Changzhou Vocational Institute of Engineering, Changzhou 213164, China; chengjin82@163.com; 3 College of Textile and Clothing, Jiangnan University, Wuxi 214122, China

**Keywords:** laser-induced irradiation, flexible printed circuit board, electroless plating, copper pattern

## Abstract

The generation of a flexible printed circuit board on polymer fabrics has been a challenge over the last decade. In this work, a copper pattern was obtained on a soft substrate of filter paper/polyacrylonitrile (FP/PAN) film, where the filter paper was commercially available. The pattern of Ag particles was first produced on an Ag^+^-doped FP/PAN composite film, followed by electroless plating of copper using the metal silver particles as seeds. The in situ reduction of silver particles and the formation of the silver agglomeration pattern were induced by laser irradiation technology on the FP/PAN/AgNO_3_ composite film. A variety of characterizations indicated that the resultant copper deposition was uniform, with good conductivity properties.

## 1. Introduction

The development of high-resolution metal patterns has allowed for significant progress in applications of the microelectronics industry such as surface-mount devices and integrated circuits [1], radio frequency identification and smart cards [2], wireless sensors and temperature sensors (T-sensor) [2,3,4], super hydrophobic surfaces [5], and flexible electronics [6]. In particular, in the wearable electronics industry, the flexible circuit board is a key element for boarding microelectronic devices. To our best knowledge, the technique based on photolithography processes is one of the most used methods to produce metal patterns on hard substrates. However, the photolithography processes have some disadvantages, such as needing expensive equipment, being time-consuming, and difficulties in adapting for patterning non-planar substrates. Furthermore, pattern fabrication on soft substrates using this method is hard to process. 

In recent years, a new technology based on laser irradiation has been developed to form metal patterns on both hard and soft substrates. There are many related technologies such as laser-foaming technology [7], laser-induced periodic surface structure technology (LIPSS) [8], and other laser ablation-based technologies, like laser-induced forward transfer (LIFT) [9], laser interference lithography (LIL) [10], matrix-assisted pulsed laser evaporation (MAPLE) [11], and pulsed laser deposition (PLD) [12]. In addition, laser-induced machining can be tuned to both material properties and desired surface patterns by controlling certain parameters of the laser such as wavelength, fluency, intensity, pulse width, total photon count, and other illumination conditions [13].

Conventional electroless plating methods include pretreatment, seeding or activation, and electroplating [14]. Pretreatment can improve the interfacial adhesion between the substrate and the deposited metal [15]. Seeding or activation of the dielectric substrate is critical to subsequent successful electroless metal deposition. Herein, we demonstrate a novel method for implanting a composite film for selective electroless copper deposition using laser-induced irradiation on Ag^+^-doped filter paper/polyacrylonitrile (FP/PAN).

## 2. Experimental

### 2.1. Materials

Polyacrylonitrile (PAN, average Mw = 50,000) and sodium potassium tartrate were purchased from Sigma-Aldrich (Shanghai, China). All other chemicals were commercially obtained from Sinopharm Chemical Reagent Co., Ltd. (Shanghai, China) and used without any further purification. Commercially available filter papers with a thickness of 0.5 mm (measured using micrometer calipers) were soaked in a PAN/dimethylsulfoxide solution, followed by drying under a vacuum to prepare the FP/PAN films (with a thickness of 0.8–1.2 mm). 

### 2.2. Laser-Induced Setup

**Figure 1:**
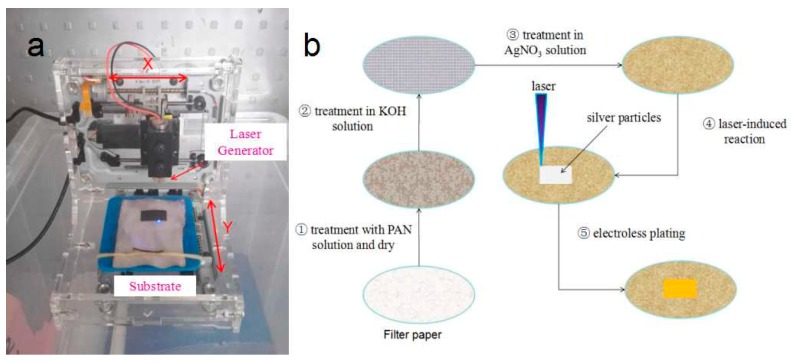
(**a**) Laser-induced set-up; and (**b**) diagram of the laser-induced and electroless plating processes.

Self-made equipment was modified from a CD/DVD optical device (λ = 405 nm) and used to provide the laser source, as shown in Figure 1a. The laser beam could be adjusted by a lens, and the focus spot size on the substrate was estimated to be 100 μm in diameter. The laser beam could be mounted by a computer-controlled x–y moving stage, which provided the relative motion of the laser beam for patterning on the composite films. In our experiments, the scanning speed could be controlled by self-developed software. The laser-induced process was operated in air at room temperature.

### 2.3. Laser-Induced and Electroless Plating

The entire process of forming a copper pattern on filter paper is similar to Chen’s report [16], as schematically shown in Figure 1b. First, the filter paper was immersed in PAN/dimethylsulfoxide solution with the PAN concentration of 0.03 g/mL for 5 min. The filter paper with the PAN solution adsorbent was then taken out and dried under a vacuum at 60 °C for 1 h to remove the dimethylsulfoxide. Thereafter, the film was immersed in KOH solution (0.5 mol/L) at 50 °C for 25 min. After rinsing with distilled water and drying, the composite film of FP/PAN was immersed in AgNO_3_ solution (100 mmol/L) at room temperature for 2 h, then rinsed again with distilled water. After drying, the Ag^+^-doped film was then placed on the laser-induced stage and fixed with a rubber band, as shown in Figure 1a. The silver ions on the desirable pattern area were irradiated by laser and reduced to metallic silver, whereas the other Ag^+^ remained in an ionic state. The film was immersed in a 1 wt % H_2_SO_4_ solution for about 15 min to exchange Ag^+^ ions with H^+^ ions. Then, the film was rinsed with distilled water and dried. Finally, the film was immersed in an electroless plating bath at room temperature for 40 min to deposit the copper patterns. The electroless plating bath consisted of NaOH (14 g/L), sodium potassium tartrate (24 g/L), CuSO_4_ (8 g/L), and 12 mL/L of formaldehyde aqueous solution (37 wt %). The deposition steps were terminated by removing the film from the bath and washing with distilled water.

### 2.4. Characterization

The topographic profiles of the filter paper films before and after laser treatment were investigated using atomic force microscopy (AFM) (Nanoscope IIIa, Digital Instrument, Santa Barbara, CA, USA) set at contact mode. Scanning electron microscopy (SEM, Hitachi 3400N, Hitachi, Japan) was applied to investigate the surface morphology of the deposited copper after coating the samples with gold. The current-voltage (I–V) characteristics of the deposited copper were measured using a direct-current voltage and a current source/monitor (Keithley, 4200-SCS, Cleveland, OH, USA).

## 3. Results and Discussion

### 3.1. Formation of Ag Particles on Filter Paper/PAN Films

The scanning speed of the laser has a great influence on the surface properties of Ag-deposited FP/PAN films. In Chen’s report [16], they developed a simple equation to analyze the relationship between the scanning speed of the pulsed laser and the laser-active area on the substrate:

(1) $$s=\frac{v}{f},$$

where *s*, *f*, and *v* represent the distance of two adjacent spots created by the laser pulse, repetition rate of pulsed laser, and the scanning speed, respectively. For the deposited silver to be connected and effective for the subsequent deposition of copper, *s* is generally smaller than the diameter of any laser spot. However, a very small value of *v* would lead to the laser pulse irradiating on the contiguous area, repeatedly, and the laser would heat this area to a very high temperature to carbonize the composite film. This equation has great potential for helping us choose a suitable scanning speed.

**Figure 2:**
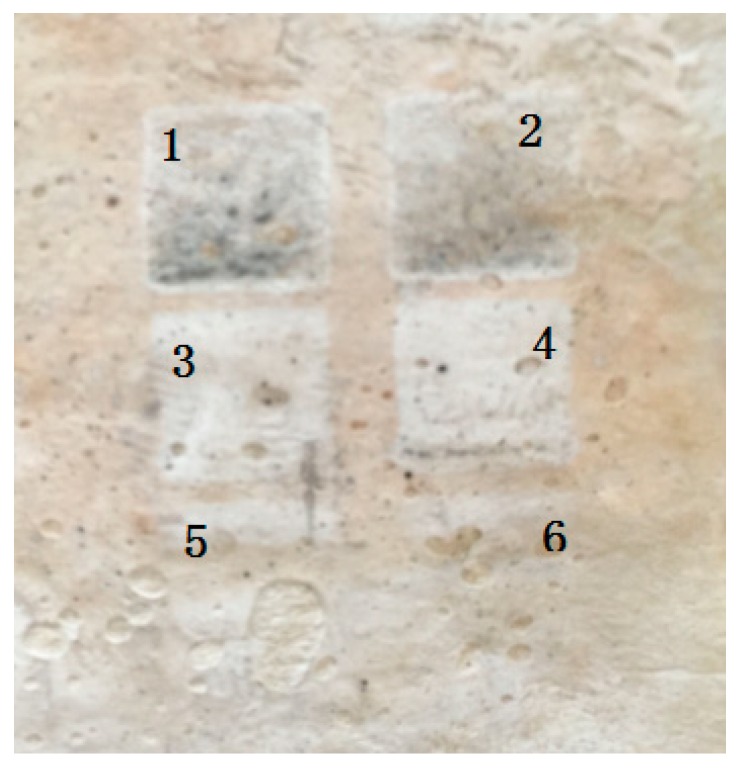
Effect of laser scanning speed on the Ag-deposited composite films of FP/PAN. Sample (**1**) 1 mm/s; (**2**) 2 mm/s; (**3**) 4 mm/s; (**4**) 6 mm/s; (**5**) 8 mm/s; (**6**) 10 mm/s.

We tried six different scanning speeds and found that the irradiation intensity increased with decreasing scanning speed. Figure 2 shows the topography of the FP/PAN films after being irradiated by a laser with various scanning speeds. It is demonstrated that a very high speed of laser scanning produced only a small amount of Ag particles on the composite film, as shown in Figure 2 (samples 5 and 6, with a scanning speed of 8 mm/s and 10 mm/s, respectively). On the other hand, if the scanning speed decreased below 2 mm/s, the Ag particle pattern became less uniform and some parts of the irradiated area turned charcoal gray, as shown in Figure 2 (samples 1 and 2). This may have been caused by the formation of Ag_2_O under the high temperature of the laser. Therefore, the most suitable scanning speed was 4–6 mm/s (samples 3 and 4 in Figure 2).

**Figure 3:**
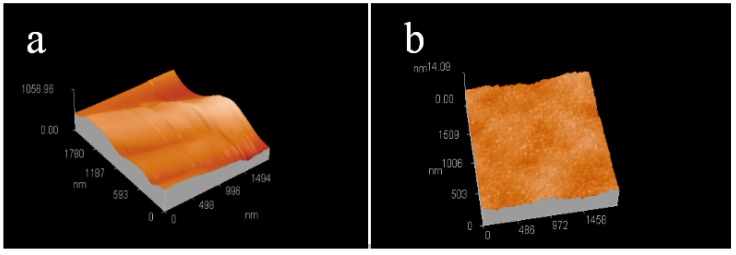
AFM images of filter-paper/PAN composite films (**a**) before and (**b**) after laser irradiation.

**Figure 4:**
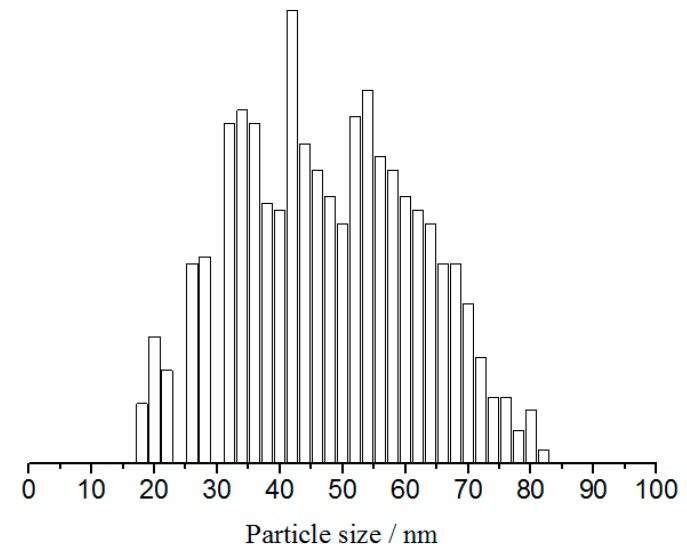
Size distribution of silver particles on the FP/PAN film after laser irradiation.

Figure 3 presents the AFM micrographs of FP/PAN composite film before and after laser irradiation. The scanning speed and fluency of laser were controlled at 5.0 mm/s and 1.0 mJ/cm^2^, respectively. It was easy to detect that the surface of the film was relatively smooth, whereas it became much rougher after irradiation by a laser due to the presence of silver particles. The silver particles agglomerated through the surface tension and resulted in a size of 40 to 80 nm, as shown in Figure 4.

### 3.2. Electroless Plating of Cu

**Figure 5:**
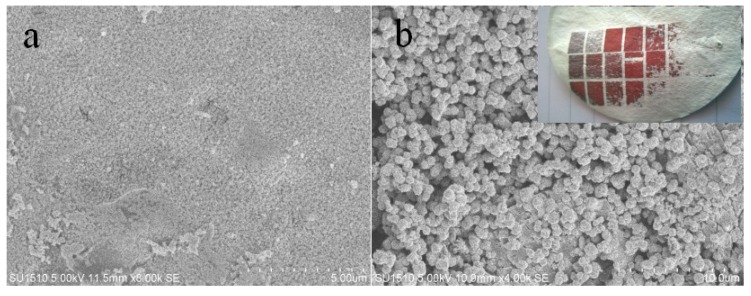
SEM images of silver particle (**a**) and copper particle (**b**) deposition on the composite films of FP/PAN. The inset photograph indicates the electroless deposited copper pattern on the composite film of FP/PAN.

**Figure 6:**
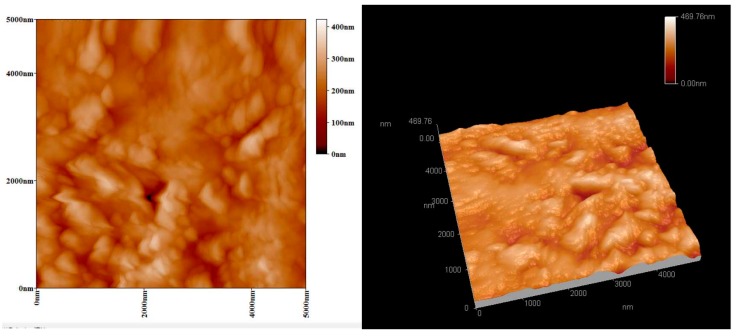
AFM images of the FP/PAN composite film after the electroless plating of copper.

The processes of forming silver particles on the surface of FP/PAN film can be described as follows: when the laser beam is focused on the film surface, the AgNO_3_ and PAN substrate absorbs the energy of the laser pulse, and a radial temperature area is formed that leads to the decomposition of PAN. The Ag^+^ is reduced by the C element from the decomposed PAN, then the reduced silver melts and agglomerates into particles at high temperature. Subsequently, the silver particles are fully covered by the copper coating during the electroless plating process. It should be noted that copper can hardly be deposited on the area without laser irradiation. Therefore, we considered that the silver particles were the active seeds for the copper plating reaction, and it was possible to produce designed copper patterns on the FP/PAN composite films. Figure 5 shows the copper patterns obtained by electroless copper plating. This demonstrated that this method yielded good selectivity to prepare metal patterns on the composite films of FP/PAN. As shown in Figure 6, the AFM image of the FP/PAN film after copper plating presented a rather rough surface. 

### 3.3. Conductivity of Deposition Copper

**Figure 7:**
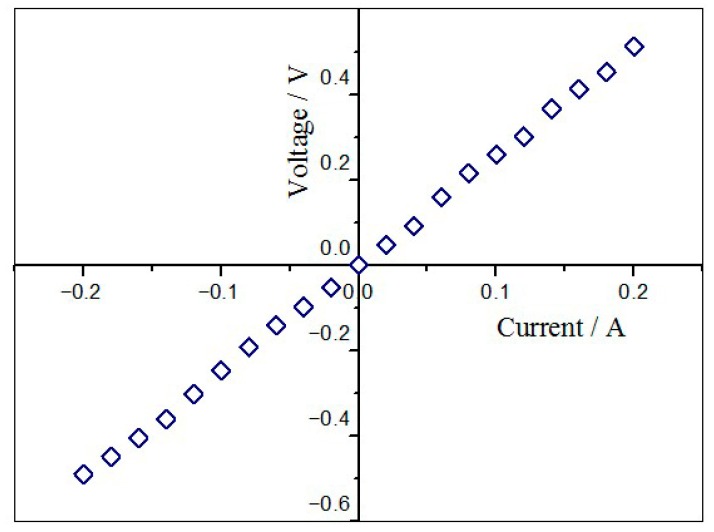
I–V curves of electroless deposited copper patterns on the composite films of FP/PAN.

With a scanning velocity of 5 mm/s, laser irradiation produced a rectangular copper film with a width of 5 mm and the length of 20 mm. Next, we coated some conductive silver paste at the margins of the rectangular copper film. After drying the silver paste under a vacuum, the electrode was clamped on the silver paste for testing the performance of I–V. Figure 7 shows the linear I–V curve, which indicated the good conductivity of electroless deposited copper patterns on the composite films of FP/PAN.

## 4. Conclusions

We reported a method using laser-induced deposition silver on FP/PAN composite films for selective electroless plating copper. The Ag^+^ iron was doped into a composite film and selectively reduced to silver particles using a pulsed laser source. Then, copper was deposited by means of electroless plating with silver particles as the seeds. The experimental results demonstrated that the scanning speed of the laser pulse greatly affected the redox reaction. The I–V curve indicated that the copper patterns on the FP/PAN composite films had good conductivity. This method provides new insight into preparing a flexible circuit board.
